# Permissibility of prenatal diagnosis and abortion for fetuses with severe genetic disorder: type 1 spinal muscular atrophy

**DOI:** 10.4103/0256-4947.72259

**Published:** 2010

**Authors:** Teguh H. Sasongko, Abd Razak Salmi, Bin Alwi Zilfalil, Mohammed Ali Albar, Zabidi Azhar Mohd Hussin

**Affiliations:** a From the Human Genome Center, School of Medical Sciences, University Sains Malaysia, Kelantan, Malaysia; b From the Department of Pediatrics, School of Medical Sciences, University Sains Malaysia, Kelantan, Malaysia; c From the Bioethics Center, International Medical Center, Jeddah, Saudi Arabia

## Abstract

Abortion has been largely avoided in Muslim communities. However, Islamic jurists have established rigorous parameters enabling abortion of fetuses with severe congenital abnormalities. This decision-making process has been hindered by an inability to predict the severity of such prenatally-diagnosed conditions, especially in genetic disorders with clinical heterogeneity, such as spinal muscular atrophy (SMA). Heterogeneous phenotypes of SMA range from extremely severe type 1 to very mild type 4. Advances in molecular genetics have made it possible to perform prenatal diagnosis and to predict the types of SMA with its potential subsequent severity. Such techniques will make it possible for clinicians working in predominantly Muslim countries to counsel their patients accurately and in harmony with their religious beliefs. In this paper, we discuss and postulate that with our current knowledge of determining SMA types and severity with great accuracy, abortion is legally applicable for type 1 SMA.

Historically the Muslim community has not permitted abortion except under exceptional circumstances strictly regulated by the consensual fatwa of religious scholars.^1^ These circumstances include cases of severe congenital abnormalities incompatible with life. Rigorous preconditions are put in place to avoid potential abuse. The preconditions are those related to the state of the disease and the diagnostic procedures involved. Factors related to the disease include certainty of death within a short period of life and poor quality of life for the family. Diagnostic and confirmative procedures include the need to involve a panel of at least three experts in establishing the diagnosis, a strict time line of 120 days (or 134 days from the last menstrual period) from conception when the termination should be done, and the requirement that the request for prenatal diagnosis and termination must come from both parents.

Islamic teachings consider the day 120 from conception as an important milestone prior to a period of ‘ensoulment’ of a developing fetus. Fetuses severely affected by conditions such as trisomy 13, 18, anencephaly, bilateral renal agenesis, Tay-Sachs disease are allowed to be terminated within this period. Abortion for serious congenital anomalies is legal in some Islamic countries such as Kuwait, Tunisia, Turkey and Jordan.^2^,^3^ However, the rulings and permissibility are complicated by the lack of a reliable predictor of seriousness and the fatality of the condition in question. This situation is made worse when faced with a genetic abnormality with a wide spectrum of clinical heterogeneity, such as spinal muscular atrophy (SMA). This paper attempts to simplify this dilemma by offering a solution to issues of predicting severity of SMA through the use of molecular genetics analysis.

## Spinal muscular atrophy

SMA is a common autosomal recessive neuromuscular disorder characterized by weakness of the proximal limb and trunk muscles. According to the disease severity and age of onset, SMA is classified into four clinical types.^4^ In 1995, Lefebvre et al^5^ demonstrated that 93% of SMA cases carry a single kind of mutation—deletion of *SMN1*, a gene located in 5q13, where all these four clinical types were mapped. Molecular analysis of SMA (i.e., identification of *SMN1* deletion) has been a method of choice in the diagnosis of the disorder^6^ and for prenatal genetic diagnosis.^7^ Molecular diagnosis of SMA has become relatively easy, inexpensive and is comfortable and accessible worldwide. Molecular analysis, including prenatal genetic diagnosis, of SMA has recently been reported in countries with a predominantly Muslim population, such as Saudi Arabia,^8^ Tunisia,^9^ Iran,^10^ Malaysia^11^ and Turkey,^12^ which indicates that Muslim countries have become largely exposed to advanced methodologies that enable easy and fast molecular diagnosis of SMA.

## SMA type 1 and fulfillment of criteria for termination of pregnancy in Muslim communities

The *fatwa*^1^ (or edict), as briefly outlined above, imposes two conditions for the eligibility for termination of pregnancy for congenital abnormalities: (1) that the condition is certain to cause death within a short period of life and (2) that the condition would result in poor quality of life for members of the family. SMA type 1 represents the most severe type of SMA. Its onset is usually during the prenatal period, immediately after birth or within 6 months of life. Weakness of the diaphragm combined with weakened intercostal muscles is the predominant cause of respiratory failure. Tongue fasciculation and weakness due to bulbar denervation cause poor sucking and swallowing, as well as decrease airway protection, increasing the risk of aspiration pneumonia, which is an important cause of mortality. Eighty percent of babies affected by SMA type 1 die by 1 year of age, 100% by 2 years, and some with extreme severity die within days of birth.^13^–^15^ The quality of life of affected patients is poor.^16^ An inability to communicate their feelings of discomfort, pain or suffering, especially in response to uncomfortable or painful medical intervention, and inability to cough, clear secretions or swallow contribute to this poor predicament.^17^ **Table 1** shows how SMA type 1 prevails in fulfilling the criteria for abortion, compared to other SMA types. These descriptions constitute conditions in which death certainly occurs within a short a period of life in all patients with SMA type 1 and where the family’s life will be significantly affected.

**Table 1:** Comparison on the extent of disability, treatment and support group among SMA types.^4^,^26^,^27^

	SMA type	I SMA type II/III/IV
Life expectance	≤ 2 years (none survived beyond 2 years of age)	Adolescence/adulthood/adulthood
Prenatal diagnosis before 120th day	Available	Available
Prenatal determination of severity before 120th day	Available	Not available
Risk to life-threaning diseases soon after birth	Respiratory insufficiency (100%)	Respiratory insufficiency (<15%)
Life productivity	None	Productive; limited physical activity
Development of self-esteem	None	Present
Availability of social support group	Limited to developed countries	Limited to develop cuntries
Availability of treatment	None prolonging life	Supportive
Independence	None	Present with limitation

## Determination of SMA severity by molecular genetic diagnosis

Fetuses with *SMN1* deletion may not manifest the expected clinical severity postnatally. As such, the challenge is to identify genetic markers that would accurately predict the clinical course after the fetus is born. This dilemma was solved by studies that showed that the SMA phenotype is directly linked to the size of the genomic deletion.^18^ Researchers have discovered disease-modifier genes—the *SMN2* and *NAIP*—that are located adjacent to *SMN1* gene within a duplicative 5q region.^19^,^20^ These observations have been demonstrated by studies conducted among Japanese, Vietnamese, Malaysian and Western ethnic populations.^19^–^24^**Table 2** shows that when *SMN1* deletion occurs concurrently with a single copy of *SMN2*, the patients always manifest the severe type of SMA, regardless of *NAIP* status. However, when two copies of *SMN2* are present in *SMN1*-deleted patients, the clinical severity is slightly variable, in which case, *NAIP* deletion status can help determine the clinical severity.

**Table 2:** Prediction of SMA severity using enotypic pattern of SMA locus.

Genotype	Severe	Milder	Total
SMN1-SMN2-NAIP^a^	Type I	Types II and III	
0-1-≥0^19^,^20^,^22^–^24^	25	0	25
0-2-0^20^,^21^,^23^	14	0	14
0-2-unknown^19^,^24^	181	15	196
0-2->0^20^,^21^,^23^	14	12	26
0->2-0^20^,^21^,^23^	0	5	5
0->2->0^20^,^21^,^23^	5	48	53

**Total**	**239**	**80**	**319**

a SMN1: 0=homozygous deletion; SMN2: 1=1 copy; 2=2 copies; > 2=more than 2 copies; NAIP: 0=homozygous deletion; > 0=no homozygous deletion.

## The diagnostic pathway for the fulfillment of criteria for termination of pregnancy in a Muslim community

Diagnostic and confirmative procedures require fulfillment of three conditions for termination of pregnancy due to congenital abnormalities: (1) the diagnosis should be confirmed by at least three experts with expertise in the related field; (2) the prenatal diagnosis and termination should be done before day 120 of conception or before the 134th day from the last menstrual period (~17 weeks); and (3) the request for prenatal diagnosis and termination must come from both parents. Specimen sampling for prenatal genetic diagnosis can be performed at 9-11 weeks of gestation from chorionic villi^25^; or at 10-12 weeks, from amniotic fluid^10^; so that the time period is sufficient to allow a complete diagnostic procedure.

**Figure 1:**
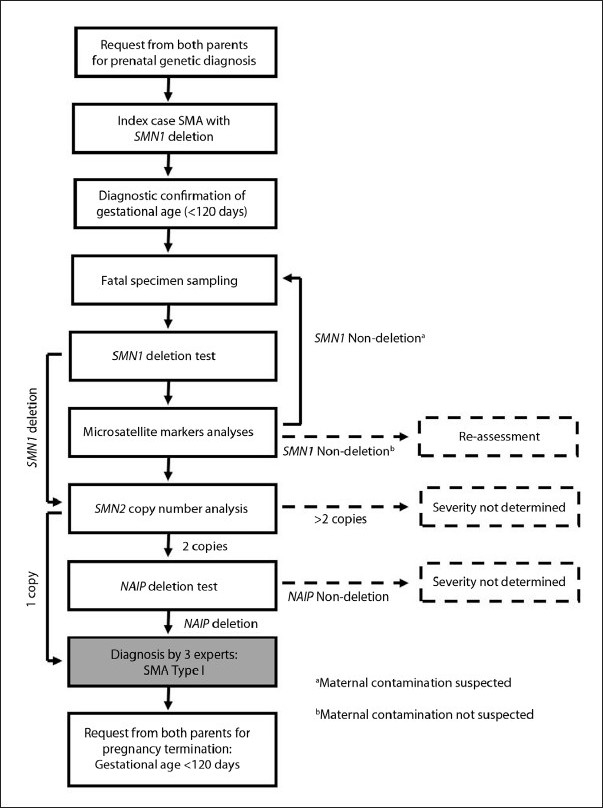
Flowchart describing the pathway of prenatal diagnostic procedures in determining SMA diagnosis and severity.

We propose a diagnostic pathway to assist clinicians in deciding on the appropriateness of termination of pregnancy once a fetus is found to carry the *SMN1* deletion. This decision-making process takes into account the copy number variation of the disease-modifying genes, which will make prediction of severity accurate, allowing termination of pregnancy within the time period permissible by jurists in the Muslim community **(Figure 1). Figure 1** outlines the flow of the diagnostic pathway we propose for determining the severity of SMA. When both parents request a prenatal genetic diagnosis, it is important to make sure that the index case is clinically SMA type 1 with deletion of the *SMN1* gene. It is also crucial to initially determine that the gestational age is younger than 120 days and that the time period is enough to allow complete diagnostic procedure.

Following fetal specimen sampling and subsequent DNA extraction, the *SMN1* deletion test is performed on the fetal DNA. Microsatellite marker analysis is performed to rule out the possibility of contamination with a maternal specimen. This is especially important in the case when no *SMN1* deletion is proven. In this case, clinical reassessment is suggested if there is no indication of maternal contamination. Re-sampling of fetal specimen is required if there is an indication of maternal contamination. If *SMN1* deletion is proven, the test would go further, into *SMN2* copy number analysis. A single copy of *SMN2* directly determines that the fetus will have a type 1 clinical severity of SMA. More than two copies of *SMN2* disable severity determination. Two copies of *SMN2* require *NAIP* deletion analysis, whereby only if the gene is deleted, can SMA type 1 be determined. Therefore, SMA type 1 can be determined based on only two genotypes (*SMN1-SMN2-NAIP*): 0-1 or 0-2-0. The diagnosis of SMA type 1 should be decided by consensus among a panel of at least three experts. Finally, termination of pregnancy should be based on a request from both parents after the final decision is made, provided the gestational age is still under 120 days.

The above diagnostic pathway should, however, be interpreted with caution in fetuses with deletion of only *SMN1* exon 8, when the *SMN1* exon 7 is found intact. This caution is of importance since only a genetic alteration within exon 7 has been proven to cause SMA.^28^ Previous studies have shown variable severity among individuals with more than one *SMN2* copies and non-deletion of *NAIP*. It is therefore not recommended to determine the severity if the fetus has a genotypic pattern other than those described within **Figure 1**.

## Conclusions and Recommendations

To the best of our knowledge, this is the first scientific approach that addresses the possibility of prenatal genetic diagnosis and abortion for congenital anomalies with high clinical heterogeneity for Muslim communities. This paper addresses an important dilemma in the Muslim community—in as much as early termination of pregnancy in case of a fatal disorder is allowed in the Islamic religion; however, previous scientific knowledge had not addressed accurately the genetic markers which are present on an unborn fetus that will accurately predict the fatality of the disease.

We have outlined the most recent findings, which indicate that the most severe type of the disease that most commonly results in fatality can in fact be diagnosed prenatally through the genomic deletion affecting *SMN1* gene and the copy number variations of disease-modifier genes, the *SMN2* and *NAIP*. Termination of such fetuses can then be permissible and done according to the laws of the Muslim community. The clinicians responsible for decision making and the parents who wish to terminate the pregnancy are therefore in a safe position, from the religious point of view. This knowledge, when made available in such communities, should lead to greater acceptability and will stand to benefit a vast number of people of the Muslim faith. It will also serve as a useful guide to clinicians working in the Muslim community and facing this dilemma.
